# Bi-allelic variants in *DAP3* result in reduced assembly of the mitoribosomal small subunit with altered apoptosis and a Perrault-syndrome-spectrum phenotype

**DOI:** 10.1016/j.ajhg.2024.11.007

**Published:** 2024-12-18

**Authors:** Thomas B. Smith, Robert Kopajtich, Leigh A.M. Demain, Alessandro Rea, Huw B. Thomas, Manuel Schiff, Christian Beetz, Shelagh Joss, Gerard S. Conway, Anju Shukla, Mayuri Yeole, Periyasamy Radhakrishnan, Hatem Azzouz, Amel Ben Chehida, Monique Elmaleh-Bergès, Ruth I.C. Glasgow, Kyle Thompson, Monika Oláhová, Langping He, Emma M. Jenkinson, Amir Jahic, Inna A. Belyantseva, Melanie Barzik, Jill E. Urquhart, James O’Sullivan, Simon G. Williams, Sanjeev S. Bhaskar, Samantha Carrera, Alexander J.M. Blakes, Siddharth Banka, Wyatt W. Yue, Jamie M. Ellingford, Henry Houlden, DDD Study, Kevin J. Munro, Thomas B. Friedman, Robert W. Taylor, Holger Prokisch, Raymond T. O’Keefe, William G. Newman

**Affiliations:** 1 Division of Evolution, Infection and Genomics, School of Biological Sciences, the University of Manchester, Manchester M13 9PL, UK; 2 Manchester Centre for Genomic Medicine, St Mary’s Hospital, the University of Manchester NHS Foundation Trust, Manchester M13 9WL, UK; 3 Institute of Human Genetics, Computational Health Center, Helmholtz Zentrum München, 85764 Neuherberg, Germany; 4 Institute of Human Genetics, School of Medicine, Technical University of Munich, 81675 Munich, Germany; 5 Université Paris Cité, Reference Center for Mitochondrial Disorders (CARAMMEL) and Reference Center Inborn Error of Metabolism, Department of Pediatrics, Necker-Enfants Malades Hospital, APHP, Filière G2M, Paris, France; 6 INSERM UMR_S1163, Institut Imagine, Université Paris Cité, Paris, France; 7 Centogene GmbH, Rostock, Germany; 8 West of Scotland Centre for Genomic Medicine, Queen Elizabeth University Hospital, Glasgow G51 4TF, UK; 9 Institute for Women’s Health, University College London, London, UK; 10 Department of Medical Genetics, Kasturba Medical College, Manipal, Manipal Academy of Higher Education, Manipal, India; 11 Service de Pédiatrie et des Maladies Métaboliques Héréditaires, Centre Hospitalier Universitaire la Rabta, Jabberi 1007, Tunis, Tunisia; 12 Laboratoire de Recherche LR12SP02, Maladies Métaboliques Héréditaires Investigations et Prise en Charge, Service de Pédiatrie et des Maladies Métaboliques Héréditaires, Centre Hospitalier Universitaire la Rabta, Jabberi 1007, Tunis, Tunisia; 13 Service de Radiologie Pédiatrique, Hôpital Robert-Debré, Assistance Publique-Hôpitaux de Paris, Paris, France; 14 Department of Medical Biochemistry and Biophysics, Karolinska Institutet, 171 65 Stockholm, Sweden; 15 Mitochondrial Research Group, Clinical and Translational Research Institute, Faculty of Medical Sciences, Newcastle University, Newcastle upon Tyne NE2 4HH, UK; 16 Department of Applied Sciences, Faculty of Health and Life Sciences, Northumbria University, Newcastle upon Tyne, UK; 17 NHS Highly Specialised Service for Rare Mitochondrial Disorders, Newcastle upon Tyne Hospitals NHS Foundation Trust, Newcastle upon Tyne NE1 4LP, UK; 18 Institute of Diagnostic Laboratory Medicine, Clinical Chemistry and Pathobiochemistry, Charité–Universitätsmedizin Berlin, Berlin, Germany; 19 Laboratory of Molecular Genetics, National Institute on Deafness and Other Communication Disorders, National Institutes of Health, Bethesda, MD 20892-3729, USA; 20 Genome Editing Unit, University of Manchester, Manchester M13 9PT, UK; 21 Newcastle University Biosciences Institute, Medical School, Framlington Place, Newcastle upon Tyne NE2 4HH, UK; 22 Genomics England, London, UK; 23 Department of Molecular Neuroscience, University College London Queen Square Institute of Neurology, London, UK; 24 Wellcome Trust Sanger Institute, Cambridge, UK; 25 Manchester Centre for Audiology and Deafness (ManCAD), School of Health Sciences, University of Manchester, Manchester, UK

**Keywords:** DAP3, mitochondria, mitoribosome, MRPS29, rare disease, Perrault syndrome, sensorineural hearing loss, ovarian insufficiency, leukodystrophy, mitoribosomal small subunit

## Abstract

The mitochondrial ribosome (mitoribosome) synthesizes 13 protein subunits of the oxidative phosphorylation system encoded by the mitochondrial genome. The mitoribosome is composed of 12S rRNA, 16S rRNA, and 82 mitoribosomal proteins encoded by nuclear genes. To date, variants in 12 genes encoding mitoribosomal proteins are associated with rare monogenic disorders and frequently show combined oxidative phosphorylation deficiency. Here, we describe five unrelated individuals with bi-allelic variants in death-associated protein 3 (*DAP3*), a nuclear gene encoding mitoribosomal small subunit 29 (MRPS29), with variable clinical presentations ranging from Perrault syndrome (sensorineural hearing loss and ovarian insufficiency) to an early childhood neurometabolic phenotype. Assessment of respiratory-chain function and proteomic profiling of fibroblasts from affected individuals demonstrated reduced MRPS29 protein amounts and, consequently, decreased levels of additional protein components of the mitoribosomal small subunit, as well as an associated combined deficiency of complexes I and IV. Lentiviral transduction of fibroblasts from affected individuals with wild-type *DAP3* cDNA increased *DAP3* mRNA expression and partially rescued protein levels of MRPS7, MRPS9, and complex I and IV subunits, demonstrating the pathogenicity of the *DAP3* variants. Protein modeling suggested that *DAP3* disease-associated missense variants can impact ADP binding, and *in vitro* assays demonstrated that *DAP3* variants can consequently reduce both intrinsic and extrinsic apoptotic sensitivity, DAP3 thermal stability, and DAP3 GTPase activity. Our study presents genetic and functional evidence that bi-allelic variants in *DAP3* result in a multisystem disorder of combined oxidative phosphorylation deficiency with pleiotropic presentations, consistent with mitochondrial dysfunction.

## Graphical abstract

**Figure:**
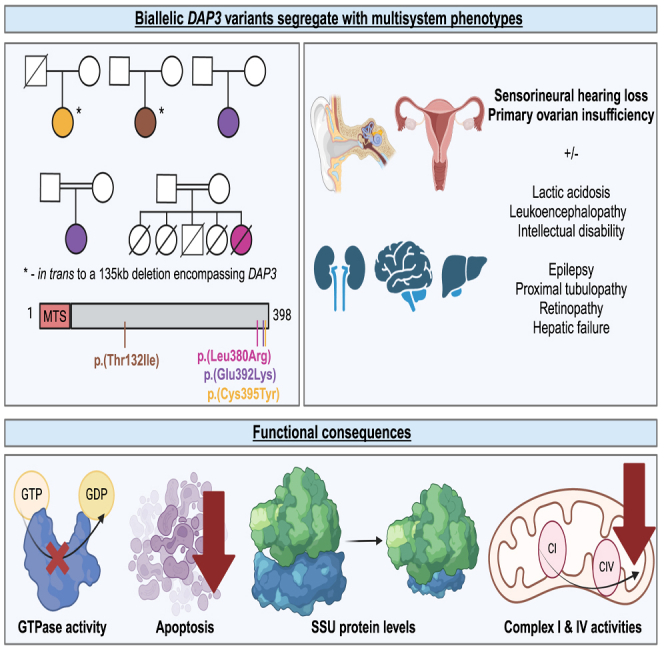

## Teaser

This study describes five unrelated individuals with bi-allelic *DAP3* variants, resulting in pleiotropic phenotypes of sensorineural hearing loss and primary ovarian insufficiency. Functional analyses using recombinant protein and fibroblasts from affected individuals revealed that *DAP3* variants can impair mitoribosome assembly, decrease protein stability, reduce GTPase activity, and diminish apoptotic sensitivity.

## Introduction

Mitochondrial ribosomes (mitoribosomes) are present in the mitochondria of all eukaryotic cells. The function of the mitoribosome is to facilitate the translation of mitochondrial mRNAs that exclusively encode components of the oxidative phosphorylation (OXPHOS) complexes. The mitoribosome consists of a small subunit (SSU) comprising 30 mitoribosomal proteins (MRPs) and a 12S rRNA that binds mRNA and tRNA to ensure accurate initiation and decoding and a large subunit (LSU) comprising 52 MRPs, 16S rRNA, and mt-tRNA^Val^, which links a nascent polypeptide to the inner mitochondrial membrane via the OXA1L insertase.^1^^,^^2^^,^^3^^,^^4^ Formation of the mitoribosome is achieved through sequential steps. For the LSU, these steps can be divided into early, intermediate, and late, whereas for the SSU, these steps are only divided into early and late.^5^ Several human diseases are caused by germline variants in genes encoding mitoribosomal proteins or assembly factors^6^ (Table S1). Death-associated protein 3 (DAP3), also known as mitoribosomal small subunit 29 (MRPS29), is a GTP-binding protein (GTBP) of the mitoribosome SSU. The precise function of DAP3 within the mitoribosome remains unclear, but it is assembled into the SSU at an early stage, interacts extensively with the 12S rRNA, and may associate with components of the inner mitochondrial membrane.^5^^,^^7^ DAP3 was initially identified as a pro-apoptotic protein^8^ involved in interferon-γ-, tumor necrosis factor alpha (TNF-α)- and FAS-induced cell death.^9^ DAP3 can also influence mitochondrial fission by modulating dynamin-related protein phosphorylation, with DAP3 depletion resulting in decreased mitochondrial protein synthesis, ATP production, and autophagy.^10^ Recently, DAP3 has also been linked to the regulation of RNA editing and splicing in the context of cancer,^11^^,^^12^ further highlighting DAP3’s broad range of functions. To date, no *DAP3* (MIM: 602074) variants have been reported in association with monogenic disorders. Perrault syndrome (MIM: 233400) is an ultra-rare, autosomal recessive condition characterized by sensorineural hearing loss (SNHL) in both sexes and primary ovarian insufficiency (POI) in 46, XX karyotype females.^13^ Neurological features are present in some affected individuals, often associated with brain white matter changes.^14^ As well as being clinically heterogeneous with variable degrees of severity, progression, and age of onset of SNHL and POI in affected individuals,^15^ Perrault syndrome is remarkably genetically heterogeneous for such a rarely reported condition. To date, bi-allelic variants in eight genes have been definitively associated with Perrault syndrome (Table S2). However, bi-allelic variants in other genes, including *RMND1* (MIM: 614917), *PEX6* (MIM: 601498), *MRPS7* (MIM: 611974), and *MRPL50* (MIM: 611854),^16^^,^^17^^,^^18^^,^^19^ have been identified in individuals with some features of Perrault syndrome; a blended phenotype accounts for some diagnoses.^20^ Despite this rich genetic architecture, potentially up to 50% of individuals with Perrault syndrome do not have a molecular diagnosis. Similarly, a large fraction of individuals with a suspected mitochondrial disease remain without a molecular diagnosis even after genome sequencing. Here, we present five individuals, each with bi-allelic variants in *DAP3* (Table 1), and accompanying functional data providing evidence that *DAP3* variants result in decreased protein stability, reduced apoptotic sensitivity, and impaired mitoribosomal assembly, which in turn lead to deficits consistent with mitochondrial disease. This study further underscores the importance of mitoribosomal proteins in auditory and ovarian function.

**Table 1:** Phenotypic summary of individuals with *DAP3* variants identified in this study

	**Proband F1**	**Proband F2**	**Proband F3**	**Proband F4**	**Proband F5**
Sex	female	female	female	female	female
Origin	UK	UK	Tajikistan	Tunisia	India
Genotype (GenBank: NM_004632.4)	c.1184G>A; 135 kb del	c.395C>T; 135 kb del	c.1174G>A; 1174G>A	c.1174G>A; 1174G>A	c.1139T>G; 1139T>G
Amino acid change (NP_001186778.1)	p.Cys395Tyr; ?	p.Thr132Ile; ?	p.Glu392Lys; Glu392Lys	p.Glu392Lys; Glu392Lys	p.Leu380Arg; Leu380Arg
Karyotype	46, XX	46, XX	N/A	N/A	N/A
Consanguinity	–	–	N/A	+	+
Age at last assessment	48 years	20 years	19 years	8 years	6 months
Bilateral sensorineural hearing loss (SNHL)	+	+	+	+	N/A
Age at SNHL diagnosis	12 months	4 years	N/A	8 years	N/A
Severity	profound	profound	N/A	profound	N/A
Treatment	unilateral cochlear implant aged 48 years, previously bilateral hearing aids	bilateral hearing aids; bilateral cochlear implants aged 20 years	N/A	hearing aids	N/A
Primary ovarian insufficiency (POI)	+	+	+	N/A	N/A
Presentation	primary amenorrhea	primary amenorrhea	primary amenorrhea	N/A	N/A
Age at POI diagnosis	14 years	14 years	19 years	N/A	N/A
Lactic acidosis	–	+ (childhood)	N/A	+ (2 years)	+
Hypoglycemia	–	+ (childhood)	N/A	–	N/A
Brain MRI	normal	normal	N/A	diffuse leukodystrophy	normal
Epilepsy	–	–	N/A	+	+
Intellectual disability	–	mild	mild	severe	N/A
Renal dysfunction	–	–	–	proximal tubulopathy	–
Retinopathy	–	–	–	+	–
Hepatomegaly	–	–	–	+	+
(Transient) liver failure	–	–	–	+	+
Height	148 cm (adult) [163.2 ± 6.5 cm]	N/A	N/A	107 cm (8 years) [129.5 ± 6 cm]	66 cm (6 months) [65.7 ± 2.3 cm]

Height standards are shown in brackets, obtained from the World Health Organization. N/A, not available.

## Material and methods

### Recruitment of research subjects

Individuals with clinical features of Perrault syndrome were recruited from the UK, Tajikistan, Tunisia, and India through GeneMatcher,^21^ the Deciphering Developmental Disorders (DDD) project,^22^ and Centogene (https://www.centogene.com/). Informed consent for DNA analysis was obtained from study participants according to local institutional ethics requirements. The individuals (and/or their legal guardians) recruited in this study gave informed consent for their participation. The individual research studies received ethical approval by the National Health Service Ethics Committee (16/WA/0017 and 10/H0305/83) and the University of Manchester.

### Whole-exome sequencing

Whole-exome sequencing (WES) was performed on DNA extracted from lymphocytes from individual F1:II-1. The SureSelect Human All Exon V5 Panel (Agilent Technologies) was used for library preparation, and sequencing was performed on the HiSeq 2500 (Illumina) system as previously described.^23^ Exome data for affected individuals in families F2–F4 were generated as previously described.^22^^,^^24^^,^^25^ For F5:II-5, the TWIST Human Core Exome Plus exome capture kit was used, and the Illumina platform was utilized for sequencing.

### Identification, amplification, and confirmation of the DAP3 fusion product

A 135 kb deletion encompassing *DAP3* was identified with the ExomeDepth (v.1.1.6) software package.^26^^,^^27^ Read depth was approximately 0.5 times the aggregated depth, indicating a single allele deletion. The fusion product and breakpoint region were confirmed in the F1 proband by Sanger sequencing using ABI big Dye v.3.1 (Thermo Scientific, Waltham, MA, USA) sequencing technology. Primers (Table S3) were designed to target polymorphisms that distinguish the two segmental duplications where the deletion breakpoints were situated.

### Maintenance of human dermal fibroblasts

Fibroblasts were cultured in high-glucose Dulbecco’s modified Eagle’s medium (Sigma) with 10% fetal bovine serum (Sigma) and 10 mL/L penicillin-streptomycin (Sigma) at 37°C/5% CO_2_.

### Fibroblast respiratory-chain activity assays

Respiratory-chain-complex activities were assessed in fibroblasts from affected individuals F1 and F4, as outlined previously.^28^

### RNA extraction, cDNA synthesis, and RT-qPCR

Fibroblasts were seeded into 6-well plates (Corning) and incubated at 37°C and 5% CO_2_ until approximately 90% confluent. After one phosphate-buffered saline (PBS) wash, RNA was extracted from cells via TRI-Reagent (Sigma) according to the manufacturer’s instructions. Total RNA was converted to cDNA with the GoScript (Promega) Reverse Transcription System with random hexamers (Thermo Scientific) according to the manufacturer’s instructions; all RNA concentrations were normalized to the lowest measured concentration. RT-qPCR reactions aimed at assessing 12S:16S ratios and mtDNA gene expression were performed with 2 μM primer pairs, PowerUp SYBR Green Master Mix (Thermo Scientific) and 1 μL template cDNA. Primer sequences are listed in Table S3. The StepOnePlus Real-Time PCR System (Applied Biosystems) was used for measuring fluorescence with the comparative CT reaction-cycle program. 2^−ΔΔCT^ values were calculated by the accompanying StepOnePlus v.2.3 data analysis package, normalizing to *ACTB* expression. For calculation of 12S:16S ratios, the 12S and 16S RQ values were totaled, and then the specific RQ value was divided by the total value. All reactions were run in triplicate in 96-well plates. Data were presented with GraphPad Prism 9 throughout this study.

### Expression and purification of recombinant wild-type and variant *DAP3*

Purified DNA fragments comprising truncated *DAP3* (DAP3Δ46) wild-type or disease-associated variants from amplified cDNA were inserted into the pMAL-c4X plasmid (New England Biolabs) at the multiple cloning site downstream of maltose-binding protein (MBP), alongside a C-terminal 6× His tag via NEBuilder HiFi DNA Assembly Master Mix (New England Biolabs) according to the manufacturer’s instructions. A pMAL-c4x vector containing MBP fused to only the 6× His tag was also produced for a negative control. All primer sequences for site-directed mutagenesis and mutagenic oligonucleotides are listed in Tables S3 and S4. After confirmation via Sanger sequencing (Eurofins Genomics), plasmids were transformed into Rosetta 2 (DE3) *E. coli* cells (Novagen) and cultured in Overnight Express TB medium (Novagen) at 19°C for 72 h. Pellets were resuspended in lysis/wash buffer comprising 20 mM Tris-Cl (pH 7.4), 150 mM NaCl, 0.1 mM DTT, 20 mM imidazole (Sigma), and 15% glycerol. All purified proteins were captured and separated by affinity chromatography utilizing the 6× His tag. His-tagged proteins were then eluted in lysis/wash buffer containing 250 mM imidazole. Selected fractions were then dialyzed overnight at 4°C in 20 mM Tris-HCl (pH 8), 200 mM NaCl, 2 mM DTT, and 15% glycerol. Proteins were then centrifuged at 17,000 × *g* for 10 min at 4°C, and the supernatants were frozen at −80°C.

### GTPase assays

GTPase assays were conducted with the GTPase-Glo Assay (Promega) in white, opaque 96-well plates (Greiner Bio-One) in accordance with the manufacturer’s guidelines. A final concentration of 5 μM DAP3 protein, 5 μM GTP, and 1 mM DTT was selected for use in the GTPase reaction, which ran for 1 h at room temperature. The luminescence of residual GTP converted to ATP was measured using the BioTek Synergy H1 microplate reader (Agilent) 10 min after the addition of a detection buffer, with reactions conducted in duplicate over three independent assays. Residual GTP was calculated as a percentage via a no-protein control, with an MBP-His protein control run in parallel to ensure the observed GTPase activity was DAP3 specific. Data were collected with Gen5 v.2.07 software (Agilent).

### Proteomic analysis

Fibroblasts from F1:II-1 and F4:II-1 were processed and analyzed through an established proteomics pipeline to quantify the protein levels of both DAP3 and the components of the mitoribosome and respiratory chain complexes. Two parameters of the protocol previously described^29^ have been modified: peptide fractionation was carried out with high-pH reverse-phase chromatographyinstead of trimodal mixed-mode chromatography, and tandem mass tag (TMT) labeling was carried with TMT 11-plex instead of TMT 10-plex reagent. For data normalization, quantification, and detection of aberrant protein expression, a denoising autoencoder-based approach, OUTRIDER2, was employed (termed PROTRIDER^29^).

### Apoptosis assays

Control and affected individual fibroblasts were seeded in opaque, white 96-well plates (Greiner Bio-One) at a density of 15,000 cells per well and incubated for 24 h at 37°C/5% CO_2_. Cells were treated with either 1 μM staurosporine (Cayman) for 4.5 h to induce the intrinsic apoptotic pathway, 0.05 or 0.5 μg/mL TNF-α (Sigma) in combination with 10 μg/mL cycloheximide (Cayman) for 24 h to induce the extrinsic apoptosis pathway, or suitable controls (0.01% DMSO and 10 μg/mL cycloheximide). Apoptotic activity was quantified using the Caspase-Glo 3/7 Assay System (Promega), as per the manufacturer’s instructions.

### Thermal-shift assay

A thermal-shift assay (TSA) was performed with the Protein Thermal Shift Dye Kit (Thermo Scientific) as per the manufacturer’s instructions in 96-well plateswith the StepOnePlus Real-Time PCR System. 1 μg of recombinant MBP-DAP3 protein was subjected to melt-curve analysis in triplicate; the temperature progressed from 25°C to 90°C with a 1% temperature ramp rate. For derivation of the melting temperature (T_m_), melt curves of the temperature against fluorescence intensity were plotted, and the temperature at which the peak fluorescent intensity was detected was selected. ATP (Cytoskeleton) and GTP (Promega) were diluted in 25 mM MgCl_2_ and incubated with recombinant protein for 10 min.

### Lentiviral transduction of *DAP3* cDNA

A third-generation lentiviral construct was assembled using VectorBuilder, inserting full-length *DAP3* cDNA upstream of T2A:EGFP under the control of an EF1α short form (EFS) promoter. Following confirmatory Sanger sequencing and lentiviral packaging, fibroblasts from affected individuals and controls were seeded in 12-well plates at a density of 40,000 cells per well for RNA extraction or into T25 flasks (Corning) at a density of 200,000 cells per flask for immunoblotting. Cells were immediately transduced in combination with 5 μg/mL polybrene (Sigma) and then incubated for 24 h at 37°C and 5% CO_2_. Cells were washed three times with PBS, and then the growth medium was replaced. 72 h after transduction, cells were washed three times with PBS and processed as required. Subsequent RNA extraction, cDNA synthesis, and qPCR analysis were conducted as described above.

### SDS-PAGE and immunoblotting

Cells were pelleted and lysed in 50 μL Pierce IP Lysis Buffer (Thermo Scientific) supplemented with 50× protease inhibitor cocktail (Promega) on ice and then agitated for 30 min at 4°C and centrifuged at 13,000 rpm for 15 min. Samples were mixed 1:1 with 2× SDS-PAGE sample buffer and heated to linearize protein and then run on a 4%–12% polyacrylamide gel made in-house at 180 V for 60 min alongside the Precision Plus Protein Dual Color Standards (Bio-Rad) ladder. Proteins were transferred onto a 0.45 μm PVDF blotting membrane (GE Healthcare) via a Trans-Blot Semi-Dry Transfer Cell System (Bio-Rad) for 30 min at 20 V. The membrane was washed with 1× TBS-Tween and blocked with 5% milk for 1 h with agitation. Primary antibodies specific to MRPS7 (Abcam, ab224442), MRPS9 (Abcam, ab187906), the five antibodies provided in the Total OXPHOS Human WB Antibody Cocktail (ATP5A, UQCRC2, SDHB, COXII, and NDUFB8) (Abcam, ab110411), and β-actin (ProteinTech; 20536-1-AP, 66009-1-Ig) were incubated overnight at 4°C in block with agitation. Dilutions were 1:200 (MRPS7, MRPS9), 1:500 (total OXPHOS), and 1:5,000 (β-actin), respectively. After washing, secondary antibodies were incubated with the membrane for 1 h at 1:10,000, and were as follows: IRDye 800CW goat anti-rabbit immunoglobulin (Ig)G (LI-COR, 926-32211) and IRDye 680RD goat anti-mouse IgG antibody (LI-COR, 926-68070). Blots were washed in TBS-Tween and visualized with the LICOR Odyssey FC imaging system using the 600, 700, and 800 channels. Quantification was achieved with LICOR Image Studio, and β-actin was used for normalizing the band intensities.

### Statistical analysis

Statistical analyses were accomplished with GraphPad Prism 9 (GraphPad) software, and one-way or two-way ANOVAs were performed with either Dunnett’s or Tukey’s multiple-comparisons tests where appropriate, as indicated in the figure legends. Statistical significance was defined as a *p* < 0.05, with additional levels of significance also expressed (^∗∗^*p* < 0.01, ^∗∗∗^*p* < 0.001, and ^∗∗∗∗^*p* < 0.0001).

## Results

**Figure 1:**
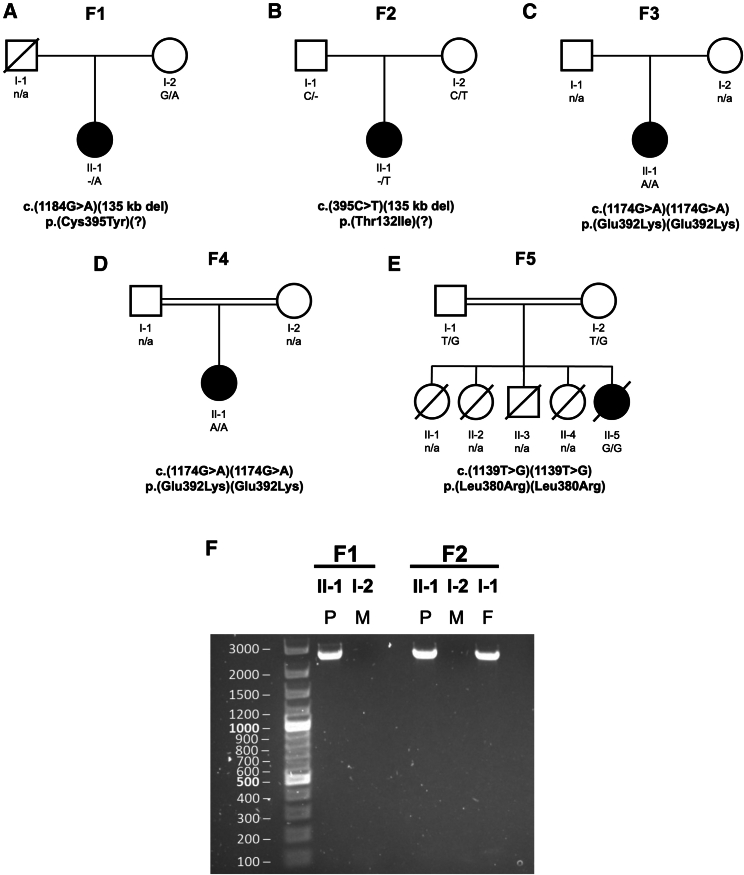
Family pedigrees and characterization of the *DAP3* deletion fusion product present in F1 and F2 (A–E) Pedigrees for the five families; known segregation and variant details are listed. All variants are annotated against the *DAP3* reference sequence GenBank: NM_004632.4. (F) PCR analysis of F1 and F2 DNA using gel electrophoresis to detect a fusion product for the 135 kb deletion. P, proband; M, mother; F, father.

No mono- or bi-allelic variants in any of the known Perrault-syndrome-associated genes were identified in any of the five affected families. Furthermore, no candidate variants in known disease-associated genes resulting in hearing loss or ovarian insufficiency were identified. We therefore proceeded to determine an alternative genetic explanation for the presentation in the affected individuals in the five families. Family F1 is a non-consanguineous British family (Figure 1A) with an affected female proband who was diagnosed with bilateral, profound SNHL at 1 year of age (Figure S1A). At age 14 years, she presented with primary amenorrhea. Her gonadotropin levels were elevated, and her karyotype was 46, XX. Subsequent investigations revealed a small vestigial uterus and streak ovaries with no follicles, leading to a diagnosis of Perrault syndrome. Otherwise, she had normal development and intellect. She had a successful unilateral cochlear implant at 48 years of age and recently presented with progressive late-onset ataxia, but an MRI scan revealed no white matter changes. The mother is unaffected, while the father is deceased from an unrelated condition. WES initially uncovered no putative pathogenic variants in known Perrault syndrome genes, but additional filtering revealed the F1 proband was compound heterozygous for the missense variant *DAP3* (c.1184G>A [GenBank: NM_004632.4] [p.Cys395Tyr]), in *trans* to a 135 kb deletion identified with multiplex-ligation-dependent probe amplification (MPLA). Breakpoints were established to recombine between g.155641696–155777755 (ClinVar: SCV005423680), which encompasses *DAP3* as well as *YY1AP1* (MIM: 607860), *GON4L* (MIM: 610393), and *MSTO2P*. WES data revealed no rare variants in these other genes. The *DAP3* variant c.1184G>A (p.Cys395Tyr) was confirmed as heterozygous in the unaffected mother by Sanger sequencing; however, it is unknown whether the deletion was a *de novo* event or inherited paternally. Family F2 is a non-consanguineous British family (Figure 1B), ascertained through the DDD study.^22^ The proband was diagnosed with bilateral SNHL at 4 years of age, which progressed to profound SNHL at high frequencies by age 20 years, at which time she had bilateral cochlear implants (Figure S1B). She presented with primary amenorrhea at 14 years, and a pelvic ultrasound scan revealed a prepubertal uterus and no visible left ovary. Endocrine tests indicated increased follicle-stimulating hormone (FSH) and lutenizing hormone (LH) levels (Figure S1C), and she received hormone replacement therapy. In early childhood, she experienced recurrent episodes of ketosis, lactic acidosis, and hypoglycemia. She also has mild intellectual disability, and her brain MRI was normal at age 19 years. Trio WES data in F2 identified a maternally inherited *DAP3* (c.395C>T [GenBank: NM_004632.4] [p.Thr132Ile]) missense variant in *trans* to a paternally inherited 135 kb deletion. A PCR fusion product of the same size as in the F1 proband was detected in the F2 proband and her unaffected father (Figure 1F). The repetitive nature of this chromosomal region made it impossible to confirm whether the breakpoints are identical in both families. Family F3 was identified through Centogene. The proband is a young woman from Tajikistan who was last assessed at 19 years of age (Figure 1C). She presented with bilateral SNHL of unknown severity, primary amenorrhea, mild intellectual disability, and developmental delay. No further clinical information is available for this family. WES revealed that the proband was homozygous for a *DAP3* (c.1174G>A [GenBank: NM_004632.4] [p.Glu392Lys]) missense variant. Family F4 is a consanguineous family of Tunisian ancestry (Figure 1D). The affected proband is a girl who presented at 15 months with neurological impairment following a febrile infection with generalized tonic and clonic seizures. Inter-ictal electroencephalogram (EEG) at 16 months demonstrated a poorly organized brain pain plot, with bilateral slow waves and no paroxysmal signs. Brain MRI revealed diffuse leukoencephalopathy, with a lactate peak on spectroscopy (Figure S2). Cerebrospinal fluid (CSF) lactate levels were normal. At 2 years, she exhibited profound SNHL, transient liver failure, and proximal tubulopathy. Electroretinogram studies revealed retinopathy. CSF and blood lactate levels were 4.5 and 5–7 mmol/L, respectively (normal ranges: 1.1–2.4 and ≤2 mmol/L), with an increased lactate/pyruvate ratio. Respiratory chain analysis activity testing on muscle cells revealed a complex IV deficiency, with borderline complex I deficiency. She was last seen at age 8 years, when she was noted to have severe intellectual disability and an unsteady gait. She was seizure free on carbamazepine. The proband was homozygous for a *DAP3* (c.1174G>A [GenBank: NM_004632.4] [p.Glu392Lys]) missense variant. Finally, family F5 is a consanguineous Indian family with a family history of neonatal and infant mortality (Figure 1E). The affected individual presented at 5 months with fever, vomiting, and lethargy. Further testing revealed hepatosplenomegaly and lactic acidemia. Brain MRI was unremarkable. No hearing evaluation was completed. The provisional diagnosis was mitochondrial disorder with hepatic failure, and she died shortly after presentation. WES revealed that the proband was homozygous for a *DAP3* (c.1139T>G [GenBank: NM_004632.4] [p.Leu380Arg]) missense variant.

**Figure 2:**
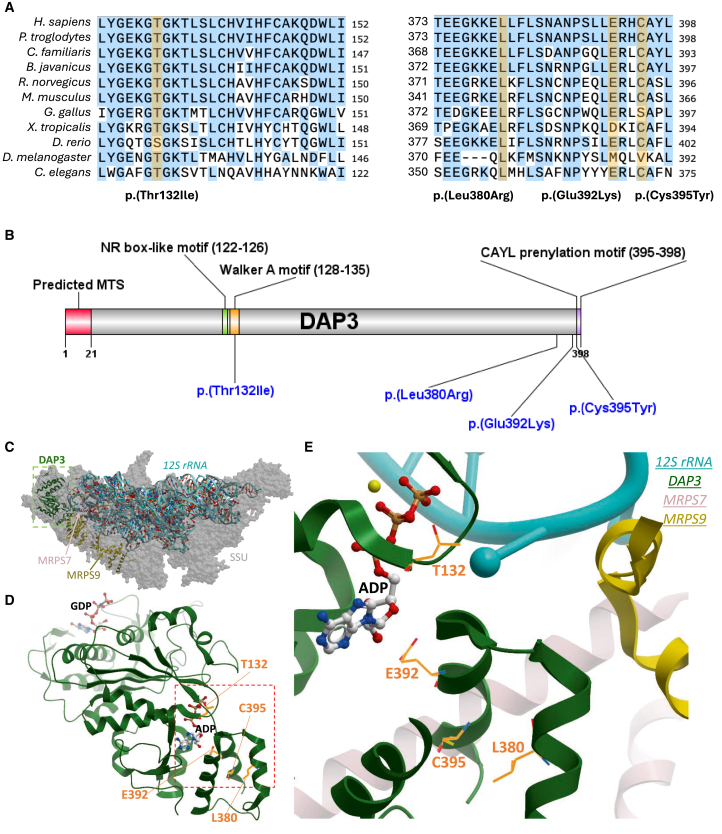
DAP3 variant residue-conservation status, variant locations, and structural context (A) Evolutionary conservation of affected DAP3 residues; a broad selection of species are highlighted. Variant amino acids highlighted in black, and yellow signifies matching to the associated human residue. Sequences were aligned via Jalview 2.11.2.7.^38^ The DAP3 reference sequences used for these species are listed accordingly: *H. sapiens* (GenBank: NP_001186778.1); *P. troglodytes* (GenBank: XP_016802675.2); *C. familiaris* (GenBank: XP_038527847.1); *B. taurus* (GenBank: NP_001106765.1); *R. norvegicus* (GenBank: NP_001011950.2); *M. musculus* (GenBank: NP_001158005.1); *G. gallus* (GenBank: XP_040546712.1); *X. tropicalis* (GenBank: NP_001016002.1); *D. rerio* (GenBank: NP_001092207.1); *D. melanogaster* (GenBank: NP_523811.1); and *C. elegans* (GenBank: AAD20727.1). (B) Overview of *DAP3* variant locations, with additional regions or domains of interest for additional context. MTS, mitochondrial targeting sequence; NR, nuclear receptor; CAYL, cysteine alanine tyrosine leucine (final four residues at the DAP3 C terminus). (C) Cryo-EM structure of human mitochondrial ribosome small subunit at 2.40 Å resolution (PDB: 7P2E), highlighting DAP3 (green), MRPS7 (rose), and MRPS9 (yellow) subunits. (D) Cartoon representation of DAP3 bound with GDP and ADP. (E) ADP binding site of DAP3 in proximity to the four sites of mutation (orange sticks).

All affected DAP3 residues are well conserved, representing 65% (p.Thr132Ile), 91% (p.Leu380Arg), 66% (p.Glu392Lys), and 73% (p.Cys395Tyr) of the respective amino acid positions across 300 orthologs using Consurf (Figure 2A). All substituted amino acids are also not present in any orthologs.^30^ Multiple *in silico* analyses predict these variants to be pathogenic or deleterious (Table S6). The four missense variants are either absent or have extremely low allele frequencies in the gnomAD v.4.0 dataset (Table S7),^31^ in further support of pathogenicity. We next inspected the site of variants at the protein level (Figure 2B), based on the recently determined structure of human mitoribosome SSUs.^32^ DAP3/MRPS29 is localized in the head region of the SSU (Figure 2C), close to the interface with the LSU. Three affected residues sit around a nucleotide-binding site, currently believed to bind ATP (Figure 2D). Threonine 132 sits within a Walker A motif (GEKGT_132_GKT), which is commonly associated with ATP or GTP/GDP binding.^33^ Cysteine 395 is located within a putative prenylation site (CAYL) at the DAP3 C terminus^34^ and is close to the interface with MRPS7, another MRP in which pathogenic variants have been associated with POI^35^^,^^36^ (Figure 2E). Glutamic acid 392 is located upstream of this prenylation site and is predicted to interact directly with ATP.^35^ Leucine 380 localizes in an α helix that packs against MRPS7 and MRPS9. To gain deeper insight into the role of DAP3 in the inner ear, we used immunofluorescence to assess DAP3 localization within the mouse organ of Corti. Since DAP3 is a component of the small mitoribosomal subunit, we used an anti-TOM20 antibody (TOM20 is a peripheral component of the translocase of the mitochondrial outer membrane complex and widely used as a robust mitochondrial marker) as a mitochondrial marker to assess the mitochondria-associated localization of DAP3. Endogenous DAP3 was identified within the murine organ of Corti but was irregularly distributed in hair cells before and after the onset of hearing, with higher levels observed in likely damaged cells, sometimes with misshapen nuclei (Figure S3A). Exogenous *DAP3*, expressed from a vector tagged with EGFP, was then transfected into the mouse organ of Corti and the vestibular sensory epithelium using a Helios gene gun to test how overexpression affected the inner ear sensory hair cells.^37^ Overexpression instigated co-localization of DAP3-EGFP with TOM20 in hair cells and diffuse staining within the cell body (Figure S3B); however, there was no discernible increase in cell death following DAP3 overexpression, indicating that compensatory mechanisms may prevent unwarranted alterations to mitoribosomal and apoptotic functions in the inner ear. We also immunostained transfected inner ear epithelial explants with DAP3 antibodies and showed that the antibody signal was increased in transfected cells only (Figure S3C) but remained nearly undetectable in non-transfected cells, indicating the specificity of the antibody to DAP3 while also pointing to very low levels of DAP3 in wild-type hair cells under normal conditions.

**Figure 3:**
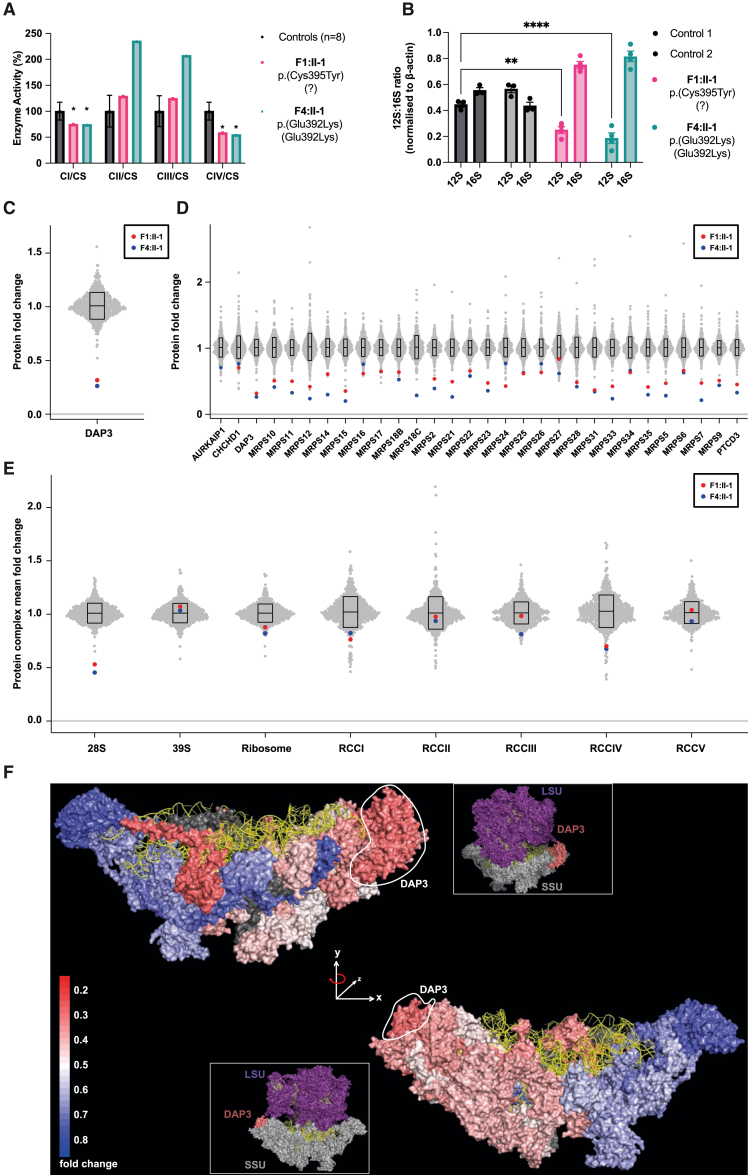
Functional and proteomic analyses of F1 and F4 proband fibroblasts reveal *DAP3* variants induce mitochondrial respiratory chain defects and decreased expression levels of small mitoribosomal subunit and OXPHOS components (A) Mitochondrial respiratory chain enzyme activities in control (black), F1:II-1 (pink), and F4:II-1 (blue) fibroblast samples. Mean enzyme activities of three protein concentrations in patient fibroblasts are compared to mean activity of three protein concentrations in control fibroblasts (*n* = 8), which are set at 100%. Error bars represent standard deviation between the controls. ^∗^ indicates enzyme activity is beyond control standard deviation values. CS, citrate synthase. (B) *MT-RNR1* (12S) and *MT-RNR2* (16S) expression levels in fibroblast cDNA. Data are expressed as a ratio using relative quantification (RQ) values. Error bars represent the SEM. *n* = 3–4, ^∗∗^*p* < 0.01 and ^∗∗∗∗^*p* < 0.0001 by two-way ANOVA with Tukey’s multiple-comparisons test; 12S RQ values of controls are compared to those of affected individuals. (C) DAP3 protein levels in affected individual fibroblasts expressed as relative *n*-fold change compared to the mean of 512 fibroblast samples. (D) Relative *n*-fold change of levels of all components of the mitoribosomal SSU in affected individual fibroblasts compared to 512 controls. (E) Grouped mean *n*-fold changes in the amount of all proteins comprising mitoribosome subunits, whole mitoribosome, and OXPHOS components are compared to the mean *n*-fold changes calculated on the basis of 512 quantitative proteome fibroblast studies. On average, we detected most of the subunits of the mitochondrial ribosome (98% of 28S and 95% of 39S) and the respiratory-chain complexes (86% of complex I, 50% of complex II, 80% of complex III, 57% of complex IV, and 81% of complex V). (F) Cryo-EM structure (PDB: 6VLZ) of mitoribosomal SSU with individual subunits colored according to their mean *n*-fold changes in abundance (of individuals F1:II-1 and F4:II-1) compared to the mean of 512 controls. Colors range from weakly reduced (blue) to strongly reduced (red), and two subunits (MRPS18C and MRPS38) are shown in dark gray because no mean *n*-fold changes could be calculated. 12S ribosomal RNA is colored in yellow, DAP3 is marked by a circle, and the small inset shows the relative position within the 55S ribosome.

To investigate the pathogenicity of the *DAP3* variants, we characterized dermal fibroblasts obtained from the affected individuals in families F1 and F4. We assessed the respiratory chain complex activities of these fibroblasts in comparison to eight healthy control fibroblasts (Figure 3A). Interestingly, fibroblasts from affected individuals F1:II-1 and F4:II-1 exhibited a reduction in both complex I (CI) and complex IV (CIV) activities compared to control reference ranges (Figure 3A; Tables S8 and S9). For both sets of fibroblasts, the CI:complexII (CII) ratio of activities was also decreased, indicative of a generalized disorder of mitochondrial translation. We also note a compensatory rise in CII and complex III enzyme activities in F4:II-1 fibroblasts, likely in response to the decrease in complex I and complex IV as described previously.^39^ Because DAP3 is a component of the small mitoribosomal subunit, we assessed whether expression of *MT-RNR1* and *MT-RNR2*, which encode 12S and 16S rRNA, respectively, was altered in F1:II-1 and F4:II-1 fibroblasts compared to healthy controls. The subsequent 12S:16S ratios were calculated with *MT-RNR1* and *MT-RNR2* relative quantification values si that specific contextual alterations to the small mitoribosomal subunit would be highlighted. The 12S component of the 12S:16S ratio was significantly reduced in F1:II-1 and F4:II-1 cDNA compared to controls, with the DAP3 p.Glu392Lys variant producing the strongest effect on *MT-RNR1* expression (p < 0.0001 [p.Glu392Lys; Glu392Lys] and 0.0019 [p.Cys395Tyr; ?]) (Figure 3B). These data indicate an impairment of mitoribosomal assembly.

**Figure 4:**
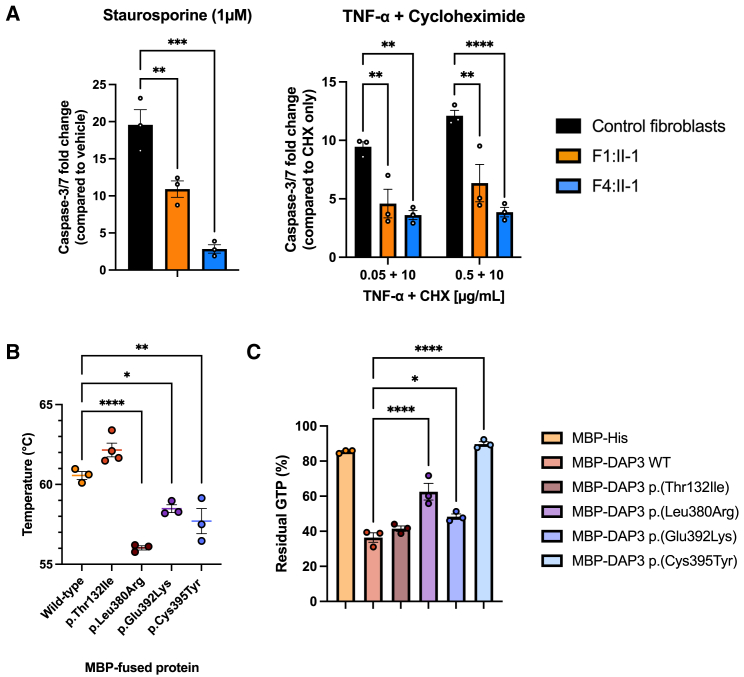
Functional analyses of fibroblasts from affected individuals and recombinant DAP3 protein establish that *DAP3* variants can diminish apoptotic sensitivity and destabilize DAP3 protein structure, impacting GTPase activity (A) Assessment of caspase-3/7 release after stimulation of intrinsic and extrinsic apoptotic pathways. Fibroblasts from affected indivudals were challenged in duplicate with staurosporine for 4.5 h or TNF-α + cycloheximide (CHX) for 24 h before the addition of assay reagent. Data are expressed as the *n*-fold change in luminescence signal in comparison to that in DMSO-treated or CHX-treated fibroblasts. Error bars represent SEM. *N* = 3, ^∗∗^*p* < 0.01, ^∗∗∗^*p* < 0.001, and ^∗∗∗∗^*p* < 0.0001 according to a one-way ANOVA with Dunnett’s multiple-comparisons test (staurosporine) or two-way ANOVA with Dunnett’s multiple-comparisons test (TNF-α); fibroblasts from affected individuals are compared to control fibroblasts. (B) Thermal stability of recombinant wild-type and variant MBP-DAP3 protein. Data points represent average T_m_ of triplicate reactions. Error bars represent SEM. *N* = 3–4, ^∗^*p* < 0.05, ^∗∗^*p* < 0.01, and ^∗∗∗∗^*p* < 0.0001, according to a one-way ANOVA with Dunnett’s multiple-comparisons test comparing wild type to variants. (C) GTPase activity of recombinant wild-type and variant MBP-DAP3 protein. Data are presented as mean luminescence produced by residual GTP, and error bars represent SEM. *N* = 3, ^∗^*p* < 0.05, ^∗∗∗∗^*p* < 0.0001 according to a one-way ANOVA with Dunnett’s multiple-comparisons test comparing wild-type protein activity to that of variants.

To assess whether *DAP3* variants influence levels of DAP3, mitoribosomal subunits, or other mitochondrial proteins, we conducted proteomic analysis on fibroblasts from F1:II-1 and F4:II-1 and compared the data to a cohort of 512 individuals to visualize outliers. Interestingly, DAP3 was reduced to approximately 25% of mean levels in fibroblasts from both affected individuals while also displaying the lowest DAP3 levels compared to any other individual in the dataset (Figure 3C). There was a remarkably consistent decrease in levels across all proteins constituting the small mitoribosomal subunit complex (Figure 3D) in fibroblasts from both affected individuals compared to the cohort, unless the protein was undetected in the mass spectrometry analysis (Table S10). When summing up the SSU and LSU overall, the two individuals with *DAP3* variants show the lowest SSU levels across the full cohort of samples, while the levels of the LSU were not affected (Figure 3E). The analysis of mitochondrial respiratory chain complexes revealed a reduction of complex I and complex IV subunits in both affected individuals. Moreover, F4:II-1 also displayed a reduction in complex III. This reduction agrees with the enzymatic analysis and reflect the downstream consequences on the translation of mtDNA-encoded respiratory chain complex subunits. To visualize subunit protein abundance in the context of its three-dimensional (3D) structure, the data were mapped onto the cryoelectron microscopy (cryo-EM) structure of the SSU (Figure 3F). Generally, proteins situated near DAP3 in the SSU are less abundant, with subcomplex formation more likely if situated on the opposite side to DAP3. These findings demonstrate independent evidence that *DAP3* variants impair assembly of the mitoribosomal SSU, impacting mitochondrial translation. To assess whether disease-associated variants affect apoptosis, we cultured fibroblasts from F1:II-1 and F4:II-1 and challenged them with common effectors of intrinsic and extrinsic apoptosis pathways. We measured caspase-3 and caspase-7 activities with a commercial luminescence-based assay. Treatment with both staurosporine and TNF-α + cycloheximide significantly reduced caspase-3/7 release in affected individual fibroblasts compared to controls (Figure 4A). The fibroblasts from F4:II-1 exhibited a stronger apoptotic defect when challenged with the intrinsic activator staurosporine compared to the fibroblasts from F1:II-1. However, there were no significant differences between fibroblasts from affected individuals when treated with the extrinsic agent TNF-α.

**Figure 5:**
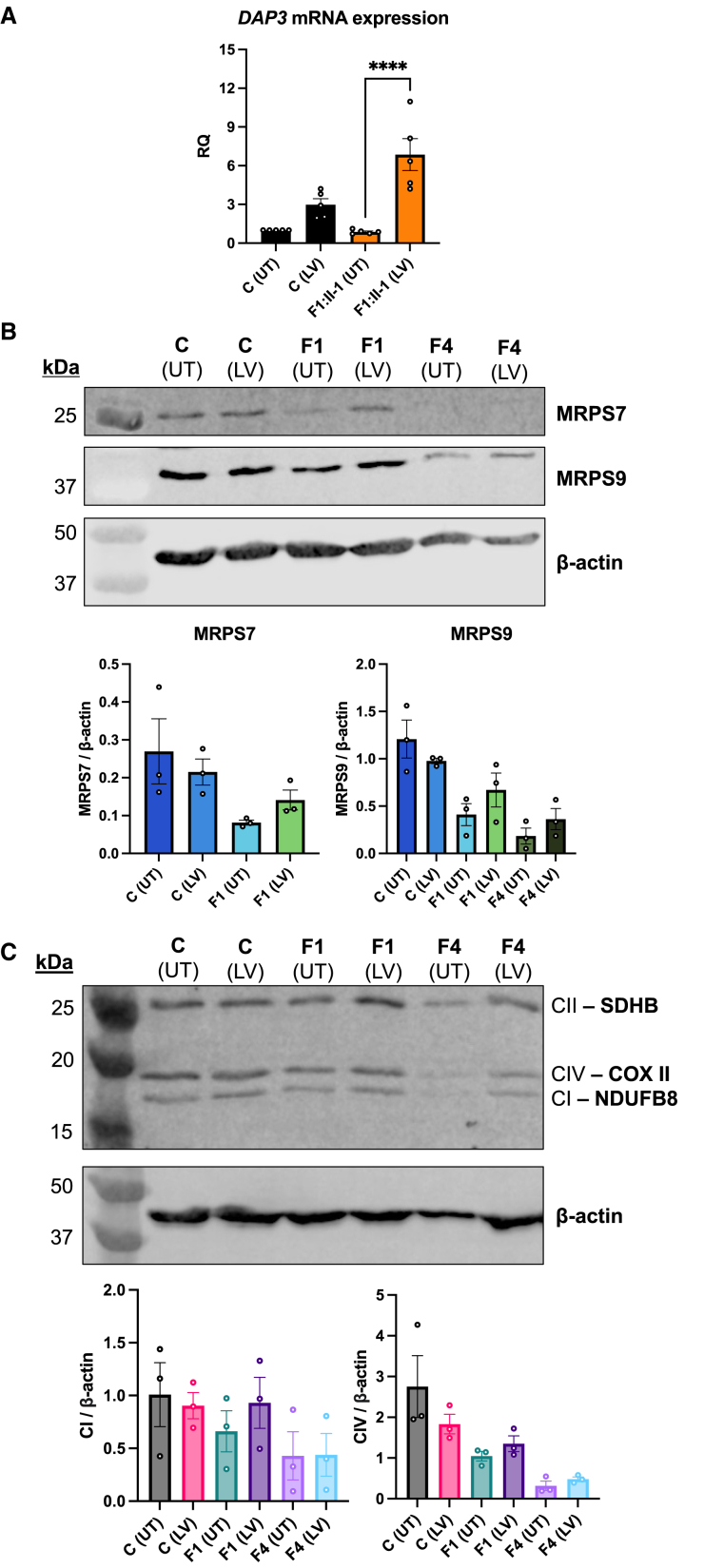
Lentiviral transduction of wild-type *DAP3* increases protein levels of MRPS7, MRPS9, and OXPHOS components in F1:II-1 and F4:II-1 fibroblasts (A) Expression of *DAP3* mRNA in control fibroblasts and fibroblasts from F1:II-1 after lentiviral transduction (LV) of *DAP3* cDNA for 72 h or untransduced (UT). Each data point represents an averaged RQ value from triplicate reactions using cDNA from independent transductions. Error bars represent SEM. *N* = 5, ^∗∗∗∗^*p* < 0.0001, one-way ANOVA with Tukey’s multiple-comparisons test. (B) Protein levels of MRPS7 and MRPS9 in control fibroblasts and fibroblasts from F1:II-1 and F4:II-1 after LV of *DAP3* cDNA for 72 h. β-actin was used as a loading control and for densitometric analysis. Blots are representative of results from three independent biological repeats. MRPS7 levels were unable to be quantified in fibroblasts from F4:II-1. (C) Protein levels of SDHB, COX II, and NDUFB8 in control fibroblasts and fibroblasts from F1:II-1 and F4:II-1 after LV of *DAP3* cDNA for 72 h. Blots are representative of results from three independent biological repeats.

To investigate the effect of *DAP3* variants on protein stability, we generated recombinant wild-type and variant DAP3 fused to MBP. TSA and subsequent melt-curve analysis highlighted a significant T_m_ decrease in p.Leu380Arg, p.Glu392Lys, and p.Cys395Tyr variants compared to the wild type (Figure 4B), indicating unfolding at lower temperatures and, consequently, reduced stability. Proteomic dissection of mitoribosomes indicated that DAP3 is the only GTBP in the SSU, suggesting secthat it could initiate or play a key role in mitochondrial protein synthesis.^10^^,^^40^ We hypothesized that *DAP3* variants could impair intrinsic GTPase activity, as disease-associated variants can impair DAP3 stability. Wild-type MBP-DAP3 exhibited GTPase activity *in vitro*. This GTPase activity was found to be significantly reduced with the DAP3 p.Leu380Arg, p.Glu392Lys, and p.Cys395Tyr variant proteins (p < 0.0001, 0.0206, and < 0.0001, respectively), correlating with the TSA data (Figure 4C). The impact of these variants was variable, with a modest increase in residual GTP observed with p.Glu392Lys compared to wild type. However, the p.Cys395Tyr variant increased residual GTP to the level observed with the negative control MBP-His, indicating low GTPase activity. Interestingly, there was no significant change in GTPase activity or thermal stability with the p.Thr132Ile variant protein. Subsequent assessment of wild-type DAP3 thermal stability after ATP and GTP treatment revealed a modest but significant increase in T_m_. which was not replicated with the p.Thr132Ile protein (Figure S4). These data suggest that DAP3 variants can reduce protein stability, subsequently impairing ligand binding and GTPase activity. To further confirm *DAP3* variant pathogenicity and the specificity of their effect, we transduced fibroblasts from F1:II-1 and F4:II-1 with a lentiviral vector expressing wild-type *DAP3* to assess whether the mitoribosomal deficit could be rescued. *DAP3* mRNA expression increased in transduced cells, as expected (Figure 5A). Basal MRPS7 and MRPS9 levels were reduced in affected individuals, concordant with proteomic analysis. After lentiviral transduction, immunoblotting also revealed a partial rescue of MRPS7 and MRPS9 protein levels in affected individual fibroblasts (Figure 5B), as well as in components of respiratory chain complex I (NDUFB8) and IV (COX II) (Figure 5C), changes that were not observed in transduced control fibroblasts.

## Discussion

Using a range of genetic, molecular, and proteomic techniques, this study reveals that bi-allelic *DAP3* variants are associated with a Perrault-syndrome-spectrum phenotype. Most known Perrault-syndrome-associated genes encode mitochondrial proteins with key roles in mitochondrial translation, which is consistent with DAP3 being a mitoribosomal SSU protein.

Phenotypes of individuals with *DAP3* disease-associated variants include a variety of features consistent with mitochondrial dysfunction; such features include lactic acidemia, neurological dysfunction, SNHL, and POI, with variable expression (Table 1). Phenotypic severity ranges from classic Perrault syndrome to childhood-onset neurological, developmental, and multisystem abnormalities. The affected individuals homozygous for the same missense variant p.Glu392Lys have markedly different phenotypic presentations, including the neurological, renal, and retinal presentations of F4:II-1. The probands from F1 and F2 have phenotypes that are less severe than those in family F4. Both individuals from F1 and F2 have a hemizygous *DAP3* missense variant in *trans* to a 135 kb deletion, consistent with the complete loss of function of one allele. Diminished respiratory chain complex activities in fibroblasts from two affected individuals are consistent with a mitochondrial translation deficit (Figure 3A). Interestingly, the fibroblasts from F4:II-1 exhibited a more pronounced respiratory chain defect, with a clear reduction in both complex I and IV activities and diminished complex I:II ratios, indicating a generalized disorder of mitochondrial translation. These data highlight that distinct *DAP3* variants have variable impacts on mitochondrial function. The mtDNA-encoded 12S and 16S rRNA are essential components of the mitoribosomal SSU and LSU, respectively. They enable protein-RNA and protein-protein interactions, which are key requirements for mitoribosome assembly and integrity.^41^ 12S:16S mRNA ratios have previously been evaluated to highlight specific discrepancies in mt-rRNA levels.^42^ 12S rRNA is associated with Perrault syndrome because of disease-associated variants in the rRNA chaperone ERAL1, which also interacts with DAP3.^42^^,^^43^ DAP3 is closely associated with the 12S rRNA, and when individual MRPs are diminished, 12S rRNA levels decline, leading to SSU assembly defects.^18^ We hypothesized that 12S rRNA levels could be reduced in fibroblasts from affected individuals, as DAP3 is assembled into the SSU at an early stage,^5^ and disrupted DAP3 function could lead to reduced mitoribosomal assembly and integrity. Indeed, 12S rRNA levels were decreased in fibroblasts, while 16S rRNA levels were unchanged, resulting in a significant alteration in the 12S:16S ratio compared to controls. In the context of total rRNA, 12S rRNA was reduced, and 16S rRNA was increased (Figure 3B). Using sensitive quantitative proteomic profiling, bi-allelic *DAP3* variants were observed to confer a profile of mitochondrial ribosomal proteins typical for an SSU deficiency. Both fibroblasts from affected individuals demonstrated a clear, specific reduction in the levels of DAP3 but also all other SSU proteins, with all LSU proteins unaffected (Figures 3C–3E). These data indicate that *DAP3* variants result in a specific impairment of SSU assembly. The loss of DAP3 could result in failure to assemble the mitoribosomal SSU, triggering degradation of the 12S rRNA and other MRPs that require DAP3 or 12S rRNA as an assembly scaffold. The generalized decrease in SSU protein levels was more evident in fibroblasts from F4:II-1, consistent with her phenotypic severity. Proteomic profiles revealed the functional consequence of impaired assembly of mitoribosome, as reduced mitochondrial translation of mtDNA encoded subunits of the respiratory chain complexes. Multiple respiratory chain complex proteins were reduced. Mainly complex I and IV mean protein abundance was affected in fibroblasts of both affected individuals, although complex III abundance was also reduced in F4:II-1, reflecting a more apparent generalized respiratory chain complex defect in this individual. The reduced respiratory chain complex activity of complex I and IV is consistent with other monogenic mitochondrial disorders^44^^,^^45^ and variants in other MRPs, including *MRPS2* (MIM: 611971), *MRPS34* (MIM: 611994), and *MRPL24* (MIM: 611836), which result in impaired mitoribosome assembly.^46^^,^^47^^,^^48^ However, despite the common molecular effects, the clinical presentation of individuals with bi-allelic pathogenic variants in MRPs is heterogeneous. Interestingly, in the fibroblasts from F4:II-1, the largest reduction was observed in MRPS7 levels. Variants in *MRPS7* have been associated with clinical features overlapping Perrault syndrome.^18^^,^^36^ DAP3 and MRPS7 are predicted to interact extensively, including at Cys395,^35^ which may explain the shared phenotypic spectrum. Variants in the gene encoding 12S rRNA (*MT-RNR1*, MIM: 561000) are associated with sensorineural, non-syndromic deafness,^49^ suggesting that, as a result of their reduced abundance, altered MRPS7 and 12S rRNA interactions might account for the SNHL in individuals with variants in *DAP3*.

We mapped mean relative changes in protein levels seen with *DAP3* variants onto a cryo-EM structure of the SSU to visualize SSU protein abundance within a structural context (Figure 3F). Interestingly, subunit abundance does not always reflect its proximity to DAP3. For example, MRPS12 and MRPS15 levels were substantially decreased in both sets of fibroblasts. Both MRPS12 and MRPS15 assemble late to the SSU and are distant from DAP3, yet both interact extensively with 12S rRNA,^5^^,^^50^ which may reflect the importance of steady-state 12S levels for successful assembly and stability. Intriguingly, four SSU proteins (MRPS7, MRPS12, MRPS15, MRPS33) exhibited marked depletion, especially in fibroblasts from F4:II-1. The 392 residue is predicted to interact with ATP, a ligand that stabilizes DAP3, and two neighboring residues, Ser389 and Arg393, also contact an unpaired base of the 12S rRNA, which may also stabilize the mitoribosome.^51^ This observation might indicate that the DAP3 p.Glu392Lys variant is more likely to induce structural defects that impair initial subcomplex assembly and reduction in SSU proteins in this individual. Taken together, these data demonstrate that *DAP3* variants effect a global reduction in SSU protein levels leading to impaired mitoribosome assembly and mitochondrial translation. Previous data have suggested that multiple Perrault-syndrome-associated genes are distinctly expressed within the spiral ganglion neurons of the cochlea, predicting that variants could interfere with auditory signal transmission.^14^ Mouse organ of Corti immunostaining did not suggest any obvious DAP3 localization patterns to specific compartments of the inner ear (Figure S3A), in contrast to Perrault-syndrome-associated protein-only RNase P catalytic subunit (PRORP), which was localized to synapses and nerve fibers of hair cells.^52^ Diffuse DAP3 cytoplasmic staining partially overlapping with mitochondrial marker TOM20 staining was observed before and after the onset of hearing in wild-type mice in some hair cells, which sometimes appeared damaged and had misshapen nuclei (Figure S3A). Exogenous overexpression shows increased mitochondrial localization in hair cells without cell damage (Figures S3B and S3C). These data imply that DAP3 is present within the mouse inner ear at relatively low levels with no clear localization profile but might be upregulated in some stress conditions, indicating that SNHL in individuals with Perrault syndrome may have diverse gene-specific etiologies. Treating fibroblasts from affected individuals with intrinsic and extrinsic apoptosis mediators revealed a decrease in apoptotic sensitivity compared to controls (Figure 4A). These data contrast with previous studies evaluating the role of DAP3 in apoptosis, which have described variable effects on extrinsic receptor-mediated cell death but no desensitizing effects reported via the intrinsic mitochondrial-mediated death mechanism.^10^^,^^53^ It is possible that *DAP3* disease-associated variants or the subsequent reduction in DAP3 abundance could affect interactions with known mediators of the intrinsic apoptosis pathway or that cells damaged by impaired mitoribosome assembly could induce non-specific mechanisms that impair the ability of the cell to detect or stimulate components of the intrinsic apoptosis pathway. DAP3 has been proposed to act as an adapter protein for death-inducing signaling complexes involved in the extrinsic pathway, recruiting fas associated death domain (FADD) to tumor-necrosis-factor-related apoptosis-inducing ligand (TRAIL) receptors (DR4 and DR5) in a GTP-dependent manner, which may be aided by DAP3-binding protein death ligand signal enhancer (DELE1).^54^^,^^55^ Diminished and unstable DAP3 protein can lead to reduced death receptor assembly, and subsequent signal transduction could explain the reduced sensitivity of fibroblasts from affected individuals to TNF-α. Melt-curve analysis revealed that DAP3 variants p.Leu380Arg, p.Glu392Lys, and p.Cys395Tyr exhibited significantly lower T_m_ than the wild type, demonstrating that these C-terminal variants destabilize DAP3 (Figure 4B). These data broadly correlate with the GTPase results, indicating that the decreased stability could diminish ligand binding and indirectly interfere with subcomplex assembly and mitochondrial protein synthesis. The DAP3 p.Leu380Arg variant conferred the most severe effect on thermal stability, consistent with the severe clinical phenotype. The p.Thr132Ile variant had no effect on thermal stability; however, treatment with ATP and GTP did not increase the melt temperature as observed with wild-type DAP3 (Figure S4). Residue 132 sits within the highly conserved Walker A motif (GxxxxGK(S/T)), which is necessary for ATP binding,^33^ indicating that the p.Thr132Ile variant likely impairs ATP binding, which subsequently reduces DAP3 stability. Proteins with GTPase activity can act as molecular switches and regulate a series of cell signaling events, including mitoribosome assembly. Mitoribosome assembly GTPases, such as ERAL1 and GTPBP10, can participate as rRNA chaperones and assembly factors as well as conduct rRNA modifications and subunit quality control.^56^^,^^57^ DAP3 is the only predicted GTPase of the mitoribosome,^40^ but the functional extent of its putative GTPase activity is unclear. A recent structural study suggested that DAP3 GTPase activity is independent of the translation cycle of the mitoribosome. However, GDP binding to DAP3 was predicted to be required for efficient mitochondrial protein synthesis via enhanced stability of the DAP3 β-hairpin at residues 208–216,^51^ highlighting the importance of DAP3 GDP binding to global mitoribosome function. We sought to understand whether recombinant DAP3 exhibited intrinsic GTPase activity and whether GTPase activity was affected by the disease-associated variants. DAP3 p.Leu380Arg, p.Glu392Lys, and p.Cys395Tyr variants significantly reduced GTPase activity, but DAP3 p.Thr132Ile had no effect (Figure 4C). These DAP3 residues are not located close to the GDP binding region, which suggests that reduced stability and improper folding may non-specifically destabilize the GDP binding pocket. Residual GTPase activity does not appear to correlate with phenotype severity, as the individual who is compound heterozygous for p.Cys395Tyr has the least severe clinical presentation. Specific variants such as DAP3 p.Cys395Tyr may also alter key DAP3 modifications, such as farnesylation of the CAYL motif.^34^ However, it is unclear whether DAP3 is sufficiently prenylated *in vivo* for this modification to contribute to phenotypic variability.^40^ We performed rescue experiments to further verify *DAP3* variant pathogenicity. Lentiviral transduction of wild-type *DAP3* increased *DAP3* mRNA expression (Figure 5A). Immunoblotting revealed that transduction increased MRPS7 levels in F1:II-1 fibroblasts and MRPS9 levels in F1:II-1 and F4:II-1 fibroblasts compared to untransduced cells but not compared to control fibroblasts (Figure 5B). This trend was not observed in control fibroblasts. The levels of CI and CIV subunits, NDUFB8 and COXII, respectively, were also partially rescued in transduced fibroblasts, particularly in F1:II-1 fibroblasts (Figure 5C). This indicates that a partial rescue of depleted mitoribosomal SSU proteins in affected individual fibroblasts might aid stability of the mitoribosomal SSU, thus partially restoring CI and CIV biogenesis. This effect has been observed in several functional studies confirming variant pathogenicity in other SSU-encoding genes, further confirming that mitoribosome destabilization is associated with various heterogeneous mitochondrial disorders.^47^^,^^48^^,^^58^^,^^59^ Collectively, these data indicate that bi-allelic *DAP3* variants result in a Perrault-syndrome-spectrum phenotype by destabilizing the mitoribosome and impairing mitochondrial translation.

Applying the ClinGen scoring criteria for gene-disease validity, we calculated a disease association score of 11, consistent with moderate evidence for disease association, which cannot be strengthened further without the identification and characterization of additional affected individuals.^60^ However, the combined genetic, clinical, and functional evidence outlined in this study provides confidence that bi-allelic *DAP3* variants are responsible for the described clinical presentations. In summary, we have identified five independent families with bi-allelic variants in *DAP3* with a pleiotropic Perrault-syndrome-associated phenotype, expanding the genetic heterogeneity of Perrault syndrome and further emphasizing the importance of mitochondrial translation in health and disease.

## Data and code availability

The *DAP3* variants were submitted to ClinVar (https://www.ncbi.nlm.nih.gov/clinvar/) (GenBank: NM_004632.4; ClinVar: SCV004228990–SCV004228993; and ClinVar: VCV003066057.1).

## Acknowledgments

We thank the families for their participation. We thank Ankur Chaurasia for support in data analysis. This study was supported by the 10.13039/501100000265Medical Research Council (MR/W019027/1 ROK, R.W.T. and W.G.N.), Action on Hearing Loss (S35 Newman); the Royal National Institute for the Deaf and the 10.13039/100014000Masonic Charitable Foundation (S60_Newman); 10.13039/501100000317Action Medical Research (GN2494); the 10.13039/100014653NIHR Manchester Biomedical Research Centre (IS-BRC-1215-20007 and NIHR203308); the Infertility Research Trust; the 10.13039/100010269Wellcome Trust ISSF pump-prime award (097820/Z/11/B); the 10.13039/501100013372Wellcome Trust Centre for Mitochondrial Research (203105/Z/16/Z to R.W.T.); the UK NHS Highly Specialised “Rare Mitochondrial Disorders of Adults and Children” Service (R.W.T.); 10.13039/501100022186The Lily Foundation (R.W.T.) through PhD studentship funding to R.I.C.G.; DBT/Wellcome Trust India Alliance for the study “Centre for Rare Disease Diagnosis, Research and Training” (IA/CRC/20/1/600002); the German Federal Ministry of Education and Research (BMBF, Bonn, Germany) and Horizon2020 through the European Joint Programme on Rare Diseases ‘GENOMIT’ (#01GM1920A), both to R.K. and H.P., and in part by the Intramural Research Program of the 10.13039/100000055NIDCD at the 10.13039/100000002NIH (DC000039 to T.B.F.). The DDD study presents independent research commissioned by the Health Innovation Challenge Fund (grant number HICF-1009-003), a parallel funding partnership between 10.13039/100004440Wellcome and the 10.13039/100004856Department of Health, and the Wellcome Sanger Institute (grant number WT098051). The research team acknowledges the support of the 10.13039/501100000272National Institute for Health Research through the Comprehensive Clinical Research Network. The views expressed in the paper are those of the authors and not necessarily those of the funders.

## Author contributions

T.B.S., R.K., L.A.M.D., A.S., H.B.T., C.B., K.T., M.O., R.I.C.G., E.M.J., A.J., I.A.B., M.B., J.E.U., J.O’S., S.G.W., S.S.B., A.J.M.B., S.C., and J.M.E. generated laboratory data. M.S., S.J., G.S.C., A.S., M.Y., P.R., H.A., A.B.C., M.E.-B., H.H., and W.G.N. contributed genetic and clinical data. T.B.S., R.K., L.A.M.D., C.B., S.B., W.W.Y., K.J.M., T.B.F., R.W.T., H.P., R.T.O’K., and W.G.N. designed and supervised the experiments and analyzed the data. T.B.S., R.T.O’K., and W.G.N. drafted the paper. All authors reviewed and critically contributed to the paper.

## Declaration of interests

The authors declare no competing interests.

## Web resources

dbSNP, https://www.ncbi.nlm.nih.gov/projects/SNP/

Ensembl Variant Effect Predictor (VEP), https://www.ensembl.org/info/docs/tools/vep/index.html

Exome Variant Server, http://evs.gs.washington.edu/EVS/

FoldX, http://foldxsuite.crg.eu/

GenBank. https://www.ncbi.nlm.nih.gov/genbank/

GeneMatcher, https://genematcher.org/

GTEx, https://gtexportal.org/home/

gnomAD, http://gnomad.broadinstitute.org/

LOVD, https://www.lovd.nl/

MutationTaster, http://www.mutationtaster.org/

OMIM, https://www.omim.org/

PolyPhen-2, http://genetics.bwh.harvard.edu/pph2/

SIFT, http://sift.bii.a-star.edu.sg.

## Supplemental information

Document S1. Figures S1–S4, Tables S2–S10, and supplemental methods

Table S1. Mitoribosomal SSU proteins grouped on the basis of stage of assembly into the SSU and their associated disorders

Document S2. Article plus supplemental information
